# Robotized unplugging of a cylindrical peg press-fitted into a cylindrical hole

**DOI:** 10.1098/rsos.230872

**Published:** 2024-01-31

**Authors:** Shuihao Xu, Duc Truong Pham, Shizhong Su

**Affiliations:** Department of Mechanical Engineering, School of Engineering, University of Birmingham, Edgbaston, Birmingham B15 2TT, UK

**Keywords:** robotized, unplugging, press-fitted, peg-and-hole, twisting-pulling, friction reduction

## Abstract

It is well accepted that remanufacturing, the returning of a product that has reached the end of its service life to its original condition, is economically and environmentally beneficial. Robotizing disassembly can make remanufacturing even more cost-effective by removing a substantial proportion of the labour costs associated with dismantling end-of-life products for subsequent processing. As unplugging of press-fitted components is a common operation in disassembly, it is appropriate to investigate how it can be robotized. This paper discusses an unplugging technique, twist-and-pull or twisting-pulling, to reduce the axial frictional resistance during the unplugging process and enable a robot to perform it easily. Through theoretical modelling, simulations, and experimental analysis, the paper explores the interaction between twisting, pulling and axial friction reduction during unplugging. Analysis of the experimental, simulation and theoretical results has confirmed that for a small radial interference, twist-and-pull reduces the axial friction and the maximum required unplugging force.

## 1. Introduction

Unsustainable consumption and manufacturing methods, particularly in developed countries, are the primary cause of the global environment's continuous deterioration [1]. In the ‘Statement on the State of the Global Climate, 2018’, the World Meteorological Organization (WMO) stated that greenhouse gases remain a global focus [2]. Greenhouse gases are a key contributor to climate change, with carbon dioxide accounting for approximately 26% of the greenhouse effect. A significant portion of the increase in carbon dioxide emissions is attributed to the waste of resources, particularly those with surplus value. As an industrial process for recovering the life of parts from end-of-life (EoL) products, remanufacturing has emerged as a significant strategy for conserving energy and protecting the environment [3]. Reusing the components retrieved from EoL products to create new products thus reducing waste and conserving resources is a desirable option [4].

Disassembly is an essential step in remanufacturing in which the scrapped products are separated into subassemblies or useable parts using manual or robotic procedures. Manual disassembly, which is time-consuming, expensive and boring, is commonly adopted in industrial remanufacturing to handle EoL products [5]. Compared with manual disassembly, robotized disassembly can improve the efficiency of disassembly [6]. For this reason, robotized disassembly, which uses intelligent manipulators to disassemble items, is being developed, and its technology needs to be promoted to reach the requirements of industrial remanufacturing [7].

The operations of disassembly can be mainly categorized into unscrewing, removing, pulling and unplugging. There has been much existing research into unscrewing, removing and pulling. For example, Apley *et al*. [8] analysed unscrewing operations and studied various methods of diagnosing faults with them. Chen *et al*. [9] designed a multi-head tool on a robot for unscrewing, drilling and grinding. Zhang *et al*. [10] developed a theoretical model based on active compliance for peg-hole disassembly. However, there are few studies related to unplugging, especially as a robotic disassembly operation. This paper focuses on single-cylinder unplugging, which is the most common among unplugging processes, and discusses an unplugging strategy suitable for robotic performance. The paper presents a new disassembly strategy, twisting-pulling, to reduce the axial frictional resistance during the process of unplugging a cylindrical pin from a cylindrical hole and enable a robot to undertake the task with less effort.

Because the authors could not find articles directly relevant to unplugging as a disassembly task other than the aforementioned publication by Zhang *et al*., a brief review was instead conducted of previous work that is partially related to this research and that provided the backdrop to the current investigation. The area focused upon was that of peg-hole insertion which is almost the reverse of unplugging and which has been investigated by many researchers over the past six decades. For example, McCallion *et al.* [11] presented a simple solution to the problem of inserting a peg in a hole. Whitney [12] analysed the process of peg-hole assembly, including approach, chamfer crossing, one-point contact and two-point contact. The idea of compliance motivated the work presented by Zhang *et al*. [10]. Liu *et al*. [13] provided a new strategy, the screw peg-hole insertion method, for axial friction reduction. Although the present authors had started this investigation prior to reading Liu *et al*.'s work, clearly, there is a relationship between the two studies, with one focusing on assembly and the other on disassembly.

Unplugging occurs when the peg and the hole are in an interference fit throughout the whole process. Goel [14] provided a mathematical analysis of an interference-fit pin joint for the initial contact force. Zhang *et al*. [15] concentrated on interference fit in ring gear-wheel couplings. The analysis results from finite-element modelling (FEM) were more accurate than those based on the thick-wall cylinder theory. Sen & Aksakal [16] indicated that the interference size affects the stresses, strains and their distributions. The increase in interference size leads to an increase in plastic deformation in the hub, but no plastic deformation occurs in the shaft (shaft-hub model). Lewis *et al*. [17] measured the interface pressure (contact force) in an interference fit using ultrasonic equipment. Lanoue *et al*. [18] mentioned the formula for the nominal contact pressure in an interference fit and fatigue strength tests by using FEM. Croccolo & Vincenzi [19] developed a Lamé-based mathematical model that applies to an axially symmetric system. They also validated the findings of the mathematical model with FEM. Croccolo *et* *al*. [20] investigated the axial pushing force in interference fit connections of various materials based on the preceding research, and the static coefficient of friction and coupling stress were computed. Paredes *et al*. [21] used Abaqus [22] to analyse the behaviour of an interference fit fastener. The findings demonstrated that the proposed analytical formula (Lamé's equation) can be used to evaluate the ‘axial loss of load from measured axial strain’. The FEM results also showed that the peak of the pressure appeared at each contact edge. Shen *et al*. [23] considered the interference fit during disassembly and indicated that various interference sizes, shaft diameters, wall thicknesses and mating lengths are the influencing variables of contact stress. The results revealed that the interference size is the most significant element, followed by the shaft diameter. Hüyük *et al*. [24] investigated the interference-fit pin-tube connection. Because of the substantial deformation at the interface, the element size (mesh size) at the pin-tube interface was selected to be less than 1% of the pin diameter.

Despite the apparent differences between this research and that reviewed above, the analytical models of interference-fit connections and peg-hole insertion served as references for the mechanical analysis of unplugging in this work. Indeed, the finite-element analysis of interference fit informed model establishment, contact modelling and boundary conditions setting for the present simulation of unplugging.

The remainder of the paper is organized as follows. The next section (§2) will establish a theoretical model of the unplugging process, which is similar to the peg-hole model. The impact of twisting on the axial friction during unplugging will then be described (§3). Simulation results for unplugging with and without twisting are next presented (§4), followed by an overview of the experiments conducted to validate the simulation and a presentation of the experimental results obtained (§5). The paper also includes an analysis of the errors between the experimental, simulation, and theoretical results, as well as potential causes (§6). The final section (§7) concludes the paper and suggests areas for further investigation.

## 2. Unplugging motion in robotic disassembly

**‘**Unplugging’ is disconnecting two objects by taking a male object (the plug) out of a matching female receptacle (the socket) [25]. Next to unscrewing, unplugging is the most common elementary operation in disassembly. In a ‘plug-socket’ disassembly operation, the fit type is generally a fixed fit or a press fit, both of which are interference fits relying on deformation of the mating components to give a secure connection [26].

**Figure 1.**
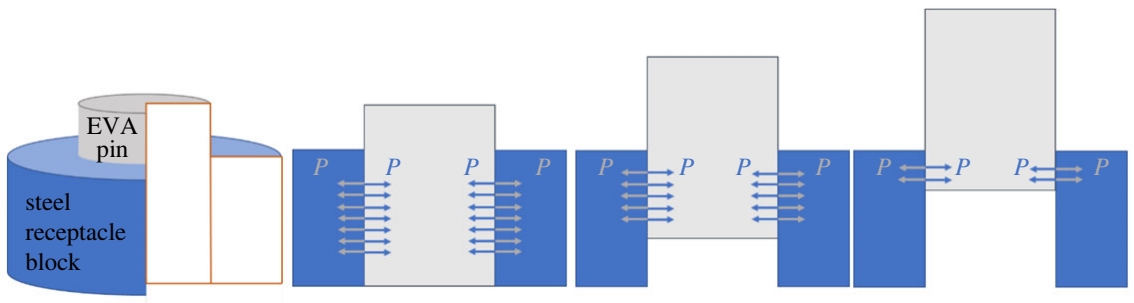
Contact force diagram in the unplugging process. EVA, ethylene vinyl acetate.

In this work, the unplugging problem has been simplified and the classic cylindrical peg-hole model has been adopted to describe the relationship between the plug and socket. The difference is that the steel peg has been replaced with a plug made of a soft material, and the unplugging motion takes place with a slight interference fit throughout (figure 1).

**Figure 2.**
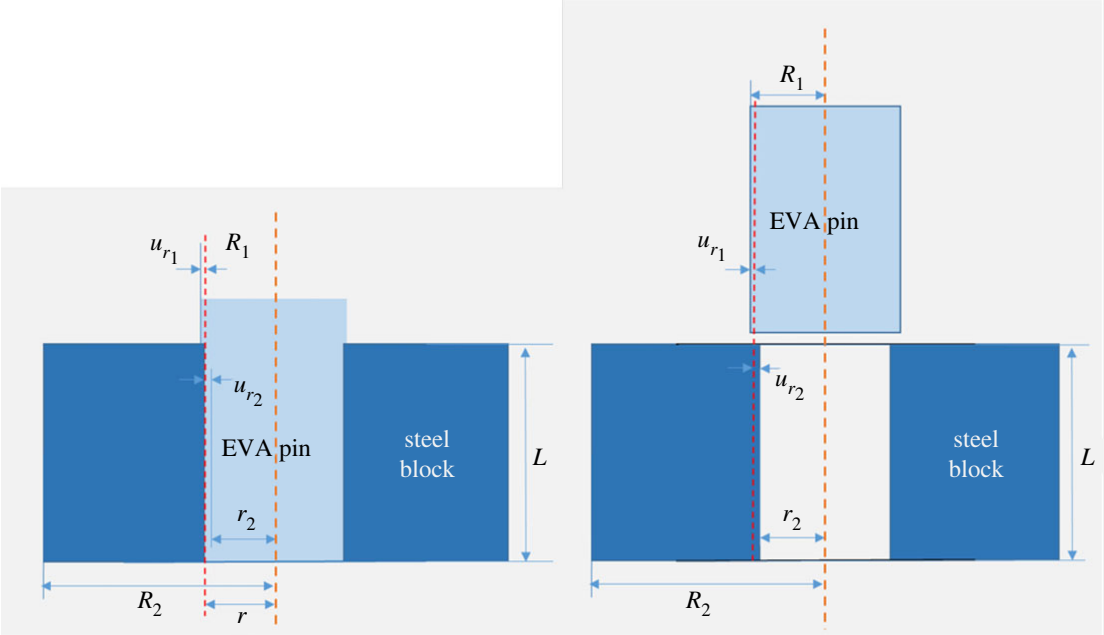
The interference-fit peg-hole model.

An interference-fit peg-hole model (figure 2) is built to study the unplugging operation [27]. In this model, in addition to the diameter of the peg being slightly larger than the inner diameter of the hole, ethylene vinyl acetate (EVA) was used as the peg material. In this theoretical model, since the radial interference (δ) is small and the material of the pin has good elasticity, only elastic deformation occurs in the pin and the hole block [28].

By using Lame's equation and thick-wall cylinder theory [29,30], the displacement of the pin ($u_{r_{1}}$) is given by:

2.1 $$\begin{array}{c} u_{r_{1}}=-\frac{P(1-v_{1})\cdot r}{E_{1}} \end{array},$$

where P is the contact pressure, r is the nominal radius and $E_{1}$ and $v_{1}$ are the Young's modulus and Poisson's ratio of EVA, respectively.

The radial displacement of the hole ($u_{r_{2}}$) is given by:

2.2 $$\begin{array}{c} u_{r_{2}}=\left(\frac{R_{2}^{2}+r_{2}^{2}}{R_{2}^{2}-r_{2}^{2}}+v_{2}\right)\frac{Pr}{E_{2}} \end{array},$$

where $r_{2}$ is the original radius of the hole, $R_{2}$ is the radius of the steel block, and $E_{2}$ and $v_{2}$ are the Young's modulus and Poisson's ratio of steel, respectively.

In this model, the interference fit size is small, and it is possible to assume in the calculation that [27]:

2.3 $$\begin{array}{c} r=r_{2}=R_{1} \end{array}.$$

The radial interference consists of the displacement of the pin and the hole:

2.4 $$\begin{array}{c} \delta =-u_{r_{1}}+u_{r_{2}} \end{array}.$$

Combining equations (2.1)–(2.4) gives the contact pressure as:

2.5 $$\begin{array}{c} P=\frac{\delta }{\frac{(1-v_{1})r_{2}}{E_{1}}+\left(\frac{R_{2}^{2}+r_{2}^{2}}{R_{2}^{2}-r_{2}^{2}}+v_{2}\right)\frac{r_{2}}{E_{2}}} \end{array}.$$

Substituting equation (2.5) into equations (2.1) and (2.2), the displacement of the pin and the hole can be expressed as:

2.6 $$\begin{array}{c} u_{r_{1}}=-\frac{\delta }{1+\frac{E_{1}}{E_{2}(1-v_{1})}\left(\frac{R_{2}^{2}+r_{2}^{2}}{R_{2}^{2}-r_{2}^{2}}+v_{2}\right)} \end{array}$$

and

2.7 $$\begin{array}{c} u_{r_{2}}=\frac{\delta }{1+\frac{E_{2}(1-v_{1})(R_{2}^{2}-r_{2}^{2})}{E_{1}[R_{2}^{2}+r_{2}^{2}+v_{2}(R_{2}^{2}-r_{2}^{2})]}} \end{array}.$$

After obtaining the contact pressure P from equation (2.5), the total contact force can be calculated from:

2.8 $$\begin{array}{c} F=2\pi rLP \end{array},$$

where 2πrL is the total contact area before unplugging.

Hence, the maximum axial resistance friction can be obtained:

2.9 $$\begin{array}{c} Rf_{\max}=\mu_{s}F=\mu_{s}2\pi rLP \end{array},$$

where $\mu_{s}$ is the coefficient of static friction between the EVA and steel.

**Figure 3.**
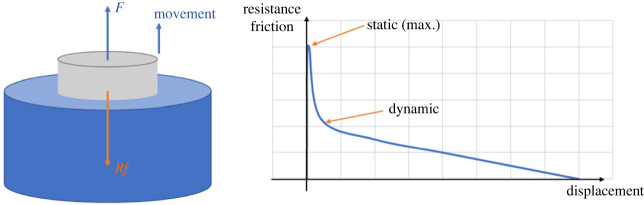
Schematic diagram of unplugging and friction force diagram in the theoretical model.

The system transforms from static to dynamic as soon as the pin begins to move. The axial resistance friction decreases rapidly because the dynamic friction coefficient is smaller than the static friction coefficient [31]. Then, as the pin is gradually pulled out, the contact area shrinks, resulting in lower friction resistance. Figure 3 demonstrates the schematic diagram of unplugging and the relationship between the frictional resistance and the displacement of the pin.

At the beginning of the extraction process, a large force is required to resist static friction, which could exceed the capacity of the robot. Consequently, if there is a method to lower the maximum friction in the axial direction, the burden on the robot will be reduced.

## 3. Combined twisting-pulling

**Figure 4.**
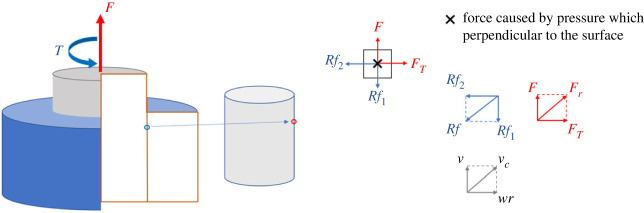
The mechanics of twisting-pulling.

Twisting is a manoeuvre often adopted by people in the process of unplugging. This means that both axial force and torque are applied on the plug to make it rotate and move up at the same time. The pin is pulled out in a spiral motion, which is presumed to require less effort than in straight pulling. The simple theoretical model shown in figure 4 is built to analyse the change in the frictional resistance and explore the mechanics of this unplugging method.

In this theoretical model, a point on the surface of the pin is selected for analysis. When applying both torque and force on the pin, the force tangent to the cylindrical surface is added together with the axial force. The frictional resistance divided into two components $Rf_{1}$ and $Rf_{2}$ will oppose the applied force and torque. The overall resistance friction can be obtained as:

3.1 $$\begin{array}{c} Rf=\sqrt{Rf_{1}^{2}+Rf_{2}^{2}} \end{array},$$

where Rf denotes the overall resistance friction, $Rf_{1}$ is the axial friction force and $Rf_{2}$ is the tangential friction force. The total applied force$\;F_{r}$ is the resultant of the axial pulling force and twisting force:

3.2 $$\begin{array}{c} F_{r}=\sqrt{F^{2}+F_{T}^{2}} \end{array}.$$

Similarly, when a constant velocity v in the axial direction and a constant angular velocity w about the axis are applied to the pin, the resultant velocity of the selected point on the pin is given by:

3.3 $$\begin{array}{c} v_{c}=\sqrt{v^{2}+{\left(wr\right)}^{2}} \end{array}.$$

The axial frictional resistance in twisting-pulling is decreased compared to that of direct pulling. The percentage reduction can be calculated by (figure 4):

3.4a $$\begin{array}{c} \mathrm{axial}\;\mathrm{friction}\;\mathrm{reduction}\;(R)=\frac{Rf-Rf_{1}}{Rf}\times 100\text{\%} \end{array}$$

and

3.4b $$\begin{array}{c} R=\frac{F_{r}-F}{F_{r}}\times 100\text{\%}=\frac{v_{c}-v}{v_{c}}\times 100\text{\%} \end{array}.$$

**Figure 5.**
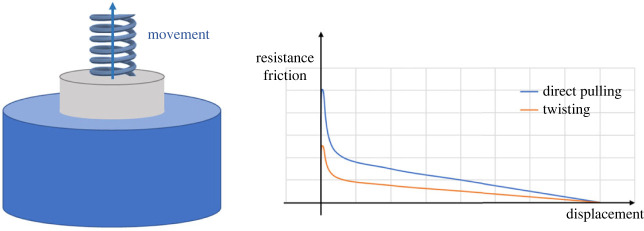
Frictional resistance in direct pulling and twisting-pulling.

Figure 5 shows the spiral trajectory of the pin and the difference in axial frictional resistance between the twisting-pulling and direct pulling techniques of unplugging. The rate at which the maximum axial frictional resistance decreases is equal to the factor R mentioned above (equation (3.4)).

## 4. Finite-element modelling of unplugging

FEM was used to simulate twisting-pulling. Abaqus/Standard 2D/3D FEM [32,33] software was adopted in this study.

In addition to the movement in the *Z* direction, the pin needs to rotate around the *Z*-axis. This is a three-dimensional problem, and therefore, a three-dimensional model was created to simulate the operation. Although unplugging is a dynamic process, static analysis was adopted to obtain the change in friction force for small movement increments.

The material parameters of the pin and the plate used in the FEM from GRANTA EduPack [34] are shown in table 1.

**Table 1.** Parameters of the material used in the simulation.

	material	Young's modulus	Poisson's ratio	density
pin	EVA	2*10^7^ Pa	0.4	0 kg m^−3^
block	steel	2*10^11^ Pa	0.25	7800 kg m^−3^

Considering the load capacity of the robot, relatively small pins were employed. Under the condition that all other parameters remain unchanged, four different sizes of pins were used to run four sets of simulations. The dimensions of the components are presented in table 2.

**Table 2.** Dimensions of the pin, hole and receptacle block in different sets of simulations.

	pin ($D_{1}$)	hole ($d_{2}$)	radial interference	block ($D_{2}$.)	depth (L)
set 1	7.02 mm	7 mm	0.01 mm	25 mm	10 mm
set 2	7.04 mm	7 mm	0.02 mm	25 mm	10 mm
set 3	7.06 mm	7 mm	0.03 mm	25 mm	10 mm
set 4	7.08 mm	7 mm	0.04 mm	25 mm	10 mm

**Figure 6.**
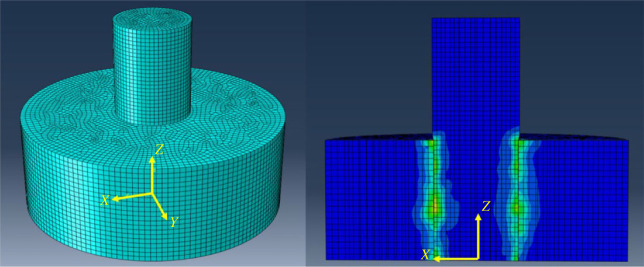
Mesh of the model and stress distribution in the components owing to the interference fit.

After building the model, two static steps were created. The first step was to apply interference fit [15,23], and the second step added displacement and rotation. Next, the interaction between the pin and the hole was set up as ‘surface-to-surface’ [18,33]. Then, according to GRANTA EduPack [34], the static and dynamic friction coefficients were set as 0.4 and 0.15, respectively. Meshing of the receptacle block was performed by applying 0.5 as the mesh size. For the hole and the pin, a finer mesh size of 0.4 was used. Figure 6 illustrates the mesh of the model and the static contact stress distribution in the pin and receptacle block caused by the interference fit.

In every set of simulations, two different conditions were applied to the pin. One was to add a linear displacement (+10 mm) in the *Z*+ direction to the pin. The other was to apply both a linear displacement (+10 mm) in the *Z*+ direction and a rotation (+π rad) in the *RZ*+ direction. In addition, the linear and angular velocities were set as constant boundary conditions.

**Figure 7.**
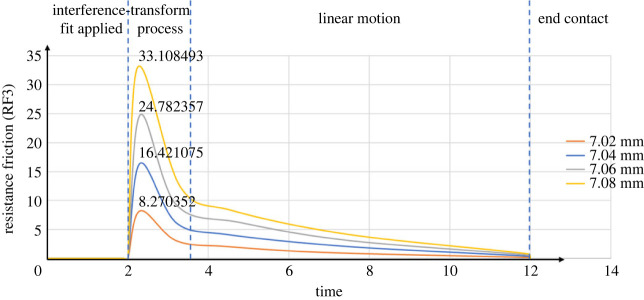
ABAQUS axial resistance friction diagrams (straight pulling, no twisting).

Figure 7 shows the values of the axial friction force changing with different pin sizes in the case of direct pulling out. With a smaller pin size, the axial resistance friction is lower throughout the process. The simulation process can be separated into four stages. In the first stage, the model only applied the interference fit, as mentioned above. No axial friction was generated at this stage. The second stage was a transformation from a static to a dynamic process. The axial resistance friction first increased substantially until the pin started to move and then decreased rapidly as the pin kept moving. This is the linear motion stage. Ideally, at this stage, the pin moved up at a constant speed, which also reduced the contact area between the pin and the hole uniformly. According to equations (2.8) and (2.9), the following equations can be obtained:

4.1 $$\begin{array}{c} S=2\pi rD \end{array},$$

where D  is the contact depth between the pin and the hole and S is the contact area.

4.2 $$\begin{array}{c} D=L-vt, \end{array}$$

4.3 $$\begin{array}{c} Rf=\mu_{d}SP, \end{array}$$

where t is time and $\mu_{d}$ is the coefficient of dynamic friction between the EVA and steel.

Combining equations (4.1)–(4.3) gives the following linear relationship between the friction force and time:

4.4 $$\begin{array}{c} Rf=\mu_{d}P*2\pi r(L-vt). \end{array}$$

The third stage is the stage in which the resistance friction decreased linearly, as characterized by equation (4.4). The final stage was when the pin was pulled out completely and there was no contact between the pin and the plate.

**Figure 8.**
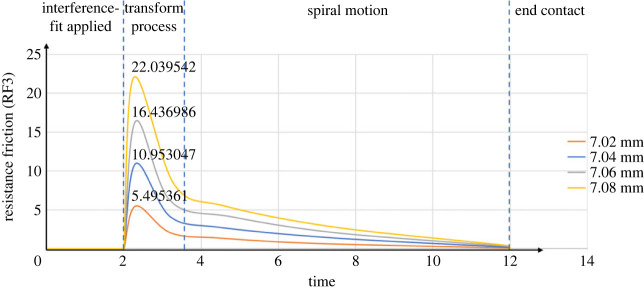
ABAQUS axial resistance friction diagrams (combined twisting and pulling).

Figure 8 presents the simulation results of the axial friction change in the case of twisting-pulling. Compared with the trends of the curves in figure 7, the axial friction changing curve trends in figure 8 are generally similar, and the process was also divided into four stages. The difference is that rotation was applied, causing the pin's movement to shift from linear to spiral. Moreover, because the tangential friction generated by rotation contributes to the resultant friction, the axial friction is reduced, lowering the highest points of the curves.

## 5. Experiments

To verify that twisting can reduce the axial friction force during unplugging, experiments were conducted that involved direct pulling and combined twisting and pulling interference-fit pins from receptacles.

**Figure 9.**
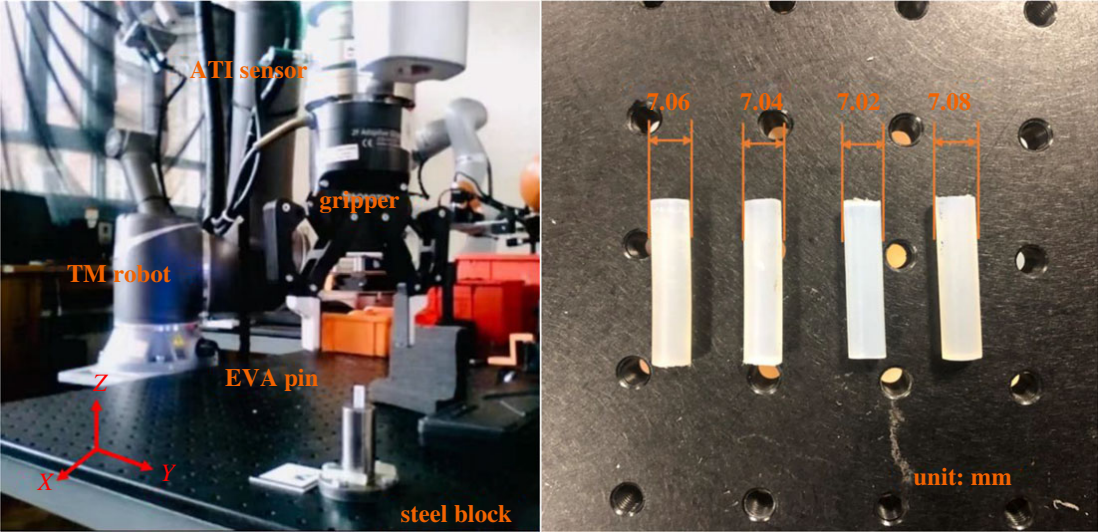
(*a*) Experimental set-up. (*b*) Four different sizes of pins used in the experiments.

In the experiments, a 6-degrees of freedom robot (TM 14) with a two-finger gripper (ROBOTIQ 2F-85) was used to grab the pin and perform the unplugging process. A 6-axis force/torque sensor (ATI axia80-m20) was installed on the wrist of the robot to record the forces and torques in real time. The experimental set-up for unplugging is shown in figure 9*a*. As in the FEM simulation, the inner diameter of the receptacle was 7 mm. Figure 9*b* shows the four different sizes of the pin (Ф7.02 mm, Ф7.04 mm, Ф7.06 mm, Ф7.08 mm), again matching the simulation conditions.

In the experiments, a Vernier calliper was used to measure the diameter of the pin at different positions, and the average value was determined as the pin's diameter. For the receptacle block, a 25 mm diameter metal cylinder with a base was used.

**Figure 10.**
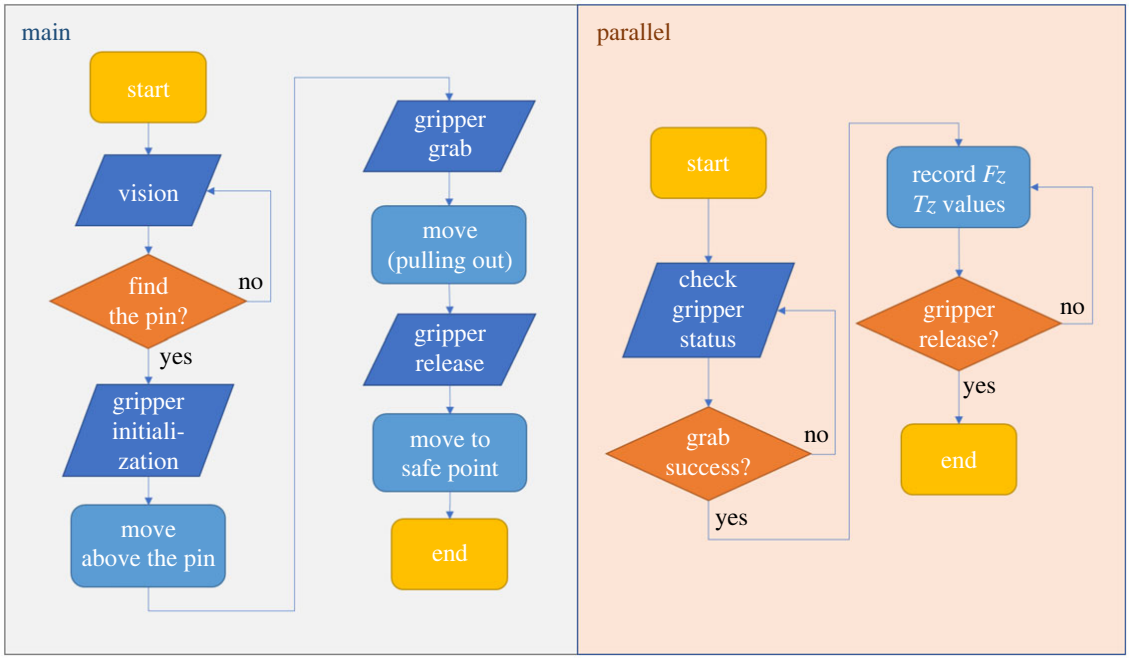
Flow charts of the robot programs.

The flow charts of the TM robot programs are shown in figure 10. The main program was used to control the robot to locate the pin with its vision system, grab it and pull it out of the receptacle block, while the parallel program recorded force/torque data during the unplugging process.

### 5.1. Direct pulling

In the first step of the experiment, the vision system on the TM robot was used to locate the pin's position. Then, the gripper clamped the pin tightly to ensure that the centre of the gripper was on the centreline of the pin. The robot moved the gripper along the *Z* + direction at a constant speed of 0.25 mm s^−1^ and stopped after moving 10 mm.

**Figure 11.**
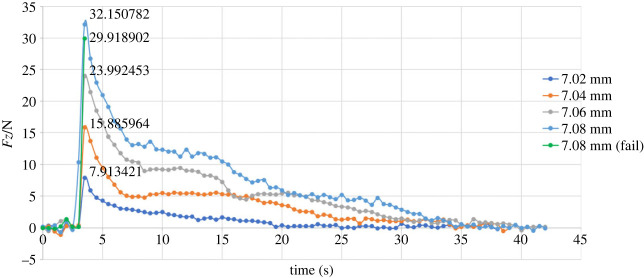
Variation in the force along the *Z*-axis (direct pulling).

Figure 11 shows the force along the *Z*-axis changing throughout the direct pulling. In this set of experiments, the 7.02 mm, 7.04 mm and 7.06 mm diameter pins were directly pulled out by the robot, and the axial friction forces in the respective experiments are shown in figure 10. However, the 7.08 mm diameter pin initially could not be pulled out of the receptacle block. Since the friction force generated by the gripper's clamping pressure was not sufficient to offset the friction between the pin and the hole, slippage occurred between the gripper and the pin. Therefore, the operation of the robot was paused, producing a force break, as shown in figure 11 (also see the electronic supplementary material). The 7.08 mm pin was subsequently bonded to the gripper, and the unplugging operation was completed successfully.

The maximum axial friction forces obtained from the theoretical model, FEM simulation and experiments are shown in table 3. Errors are defined as:

5.1 $$\begin{array}{c} \mathrm{error}=\left|\frac{\mathrm{theoretical}\;\mathrm{value}-\mathrm{simulated}\;\mathrm{or}\;\mathrm{experimental}\;\mathrm{value}}{\mathrm{theoretical}\;\mathrm{value}}\right|\times 100\text{\%} \end{array}.$$

**Table 3.** Maximum axial friction forces (direct pulling).

pin size (mm)	maximum axial resistance friction (N)		
	theoretical model	simulation (error)	experiment (error)
7.02	8.375836	8.270352 (1.26%)	7.913421 (5.52%)
7.04	16.751671	16.421075 (1.97%)	15.885964 (5.17%)
7.06	25.127507	24.782357 (1.37%)	23.992453 (4.52%)
7.08	33.503342	33.108493 (1.18%)	32.150782 (4.04%)
average error	/	1.45%	4.81%

### 5.2. Combined twisting-pulling

Combined twisting-pulling experiments necessitate the addition of the rotation of the robot end joint to the operation procedure compared with the direct pulling experiments. The constant speed of movement in the + *Z* direction was reduced from 0.25 mm s^−1^ to 0.15 mm s^−1^, and a +π rad rotation at an angular velocity of +(3π/200) rad/s was superimposed on the linear motion. Low extraction speeds were specified because of the rotational range and torque constraints on the robot joints. Another reason for selecting these speeds was to ensure that the parameters in the experiments and simulations were consistent. In addition to the axial friction force (*Fz*), the axial torque (*Tz*) was also recorded.

**Figure 12.**
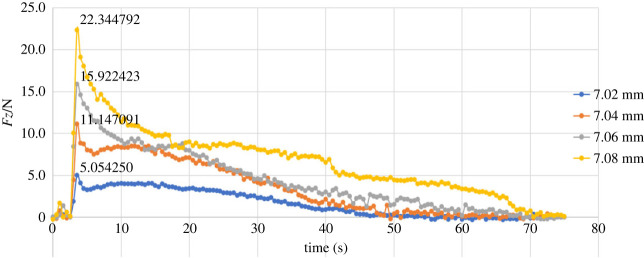
Variation in the force along the *Z*-axis (combined twisting-pulling).

**Figure 13.**
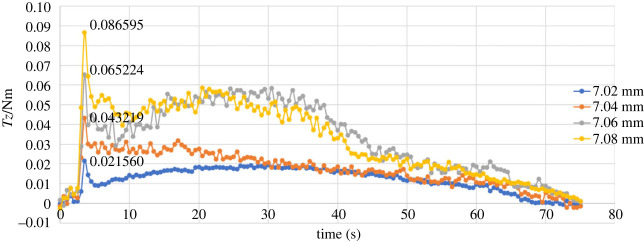
The change in the torque on the *Z*-axis in the case of twisting pulling.

Figures 12 and 13 (also see the electronic supplementary material) show *Fz* and *Tz* for different pin sizes in the case of twisting-pulling. In this group of experiments, the pins were all successfully pulled out. Compared with direct pulling, the maximum axial friction force produced by twisting-pulling was decreased. The Ф7.08 mm pin that failed to pull out in the direct pulling experiment was easily removed from the receptacle block by the twisting-pulling method. However, the latter led to large fluctuations in friction throughout this group of experiments, especially for the *Tz* curves. The reasons for the fluctuations are that the torque generated by the unplugging motion is small (less than 0.1 Nm), and the sensor used in the experiments is very sensitive. Second, owing to the use of EVA, a polymer material, the deformation and surface roughness of the pin would undergo directional and dimensional alterations as a consequence of twisting.

Table 4 gives the maximum axial friction forces obtained from the theoretical model, simulation and experiments.

**Table 4.** Maximum axial friction force (combined twisting-pulling).

pin size (mm)	maximum axial resistance friction (N)		
	theoretical model	simulation (error)	experiment (error)
7**.**02	5.635262	5.495361 (2.48%)	5.054250 (10.31%)
7**.**04	11.270524	10.953047 (2.82%)	11.147091 (1.10%)
7**.**06	16.905787	16.436986 (2.77%)	15.922423 (5.82%)
7**.**08	22.541049	22.039542 (2.22%)	22.344792 (0.87%)
average error	/	2.57%	4.53%

The axial friction reduction (R), as an important measure of whether the twisting-pulling method is effective, is shown in table 5.

**Table 5.** Axial friction reduction.

pin size (mm)	axial friction reduction (*R*)		
	theoretical model (%)	simulation (%)	experiment (%)
7**.**02	32.72	33.55	36.14
7**.**04	32.72	33.30	29.83
7**.**06	32.72	33.67	33.64
7**.**08	32.72	33.43	30.50
average *R*	32.72	33.49	32.53

## 6. Discussion

In the FEM simulation, GRANTA EduPack was employed as the reference for material selection. EVA was selected as the pin material because of its good tensile and torsional elasticity. To make the simulation closer to reality, chamfering was used in building the pin and the receptacle block. The actual maximum contact area between the pin and the hole was reduced owing to the insertion of the chamfer. As a result, the value of the maximum friction force in the simulation is slightly lower than the theoretical value, as shown in tables 3 and 4.

The plots in figure 11 show that during the first few seconds of the experiment, the values of *Fz* fluctuated slightly because of the gripper operation. After that, the values of *Fz* exhibited sudden increases of different magnitudes according to different radial interferences, and the maximum value was lower compared with the theoretical and simulated maximum values. The reasons for the error include the uneven interference fit caused by the deformation of the material, the position error between the robot gripper and the experimental model, and the trajectory and speed error of the robot motion. The reasons for the large fluctuation include the small size of the model in the experiment causing the small torque applied on the pin and the high accuracy of the sensor, which means that although the distance error and operation error are small, they can produce large numerical changes. Furthermore, the simultaneous action of tensile deformation and torsional deformation causes the pin to slide and rebound in multiple stages during the unplugging process, which is also the reason why the moment curve is jagged. Although the error of the maximum resistance friction of different sizes compared to the theoretical value varies greatly in this set of experiments, the average error is 4.53%, which is within an acceptable range. In addition, the average axial friction reduction is 32.53%, which is very close to the theoretical reduction of 32.72%.

## 7. Conclusion

This paper has studied the twist-and-pull method of unplugging a cylindrical peg press-fitted into a cylindrical hole. FEM simulations and experiments have confirmed the theoretically predicted reduction in axial friction and pulling effort when the amount of radial interference is small.

Although twisting causes the friction force to fluctuate more during the unplugging process, the overall friction in the axial direction is significantly decreased. In addition, using the same axial velocity and angular velocity to unplug pins with different amounts of radial interference results in similar axial friction reductions.

The paper has provided an improved understanding of robotized unplugging. It has introduced a novel disassembly strategy that involves twisting and pulling to reduce axial friction. This disassembly strategy enables robots to perform cylinder unplugging more easily. It can be used to extract components such as dowels, nails, bushes, etc.

There are also shortcomings in the theoretical model established in this paper as well as the disassembly strategy employed. On the theory side, a limitation of this research is that only a simplified linear-elastic model was used to verify the twisting-pulling method. While the model is applicable if there is no plastic deformation, large errors may be seen in practice when local stresses exceed the plastic threshold. On the experimental side, as the material of the peg is EVA and is much softer than that of the receptacle block which is steel, repeated tests could not be performed on the same peg to determine the effect of the twist-and-pull operation on surface wear. Moreover, the twisting-pulling method is only applicable to single-cylinder unplugging and cannot be adapted to multiple-cylinder or cuboid unplugging.

To address the above shortcomings, a theoretical model that includes both elastic and plastic deformation could be developed to handle the situation where local stresses exceed plastic thresholds. Different materials should be investigated to verify the applicability of this twisting method. Wear analysis of unplugging is necessary to determine the extent of the damage to the plug being twisted and pulled out. In addition, the development of a disassembly method that can be adapted to multiple cylinders should be considered in future work.

## Data Availability

The experiments results are uploaded as the electronic supplementary material [35]. All data in the file are from experimental measurements, collected from sensors.
